# Binocular Suppression in the Macaque Lateral Geniculate Nucleus Reveals Early Competitive Interactions between the Eyes

**DOI:** 10.1523/ENEURO.0364-20.2020

**Published:** 2021-04-05

**Authors:** Kacie Dougherty, Brock M. Carlson, Michele A. Cox, Jacob A. Westerberg, Wolf Zinke, Michael C. Schmid, Paul R. Martin, Alexander Maier

**Affiliations:** 1 Department of Psychology, College of Arts and Science, Vanderbilt Vision Research Center, Center for Integrative and Cognitive Neuroscience, Vanderbilt University, Nashville, TN 37235; 2 Princeton Neuroscience Institute, Princeton University, Princeton, NJ 09540; 3 Department of Brain and Cognitive Sciences, University of Rochester, Rochester, NY 14620; 4 Faculty of Science and Medicine, University of Fribourg, Fribourg CH-1700, Switzerland; 5 Save Sight Institute and Australian Research Council Centre of Excellence for Integrative Brain Function, The University of Sydney, Sydney, New South Wales 2006, Australia; 6 Biosciences Institute, Faculty of Medical Sciences, Newcastle University, NE2 4HH, UK

**Keywords:** binocular, interocular suppression, lateral geniculate nucleus, macaque, monocular, vision

## Abstract

The lateral geniculate nucleus (LGN) of the dorsal thalamus is the primary recipient of the two eyes’ outputs. Most LGN neurons are monocular in that they are activated by visual stimulation through only one (dominant) eye. However, there are both intrinsic connections and inputs from binocular structures to the LGN that could provide these neurons with signals originating from the other (non-dominant) eye. Indeed, previous work introducing luminance differences across the eyes or using a single-contrast stimulus showed binocular modulation for single unit activity in anesthetized macaques and multiunit activity in awake macaques. Here, we sought to determine the influence of contrast viewed by both the non-dominant and dominant eyes on LGN single-unit responses in awake macaques. To do this, we adjusted each eye’s signal strength by independently varying the contrast of stimuli presented to the two eyes. Specifically, we recorded LGN single unit spiking activity in two awake macaques while they viewed drifting gratings of varying contrast. We found that LGN neurons of all types [parvocellular (P), magnocellular (M), and koniocellular (K)] were significantly suppressed when stimuli were presented at low contrast to the dominant eye and at high contrast to the non-dominant eye. Further, the inputs of the two eyes showed antagonistic interaction, whereby the magnitude of binocular suppression diminished with high contrast in the dominant eye, or low contrast in the non-dominant eye. These results suggest that the LGN represents a site of precortical binocular processing involved in resolving discrepant contrast differences between the eyes.

## Significance Statement

A fundamental feature of the primate visual system is its binocular arrangement, which affords stereovision and hyperacuity. A consequence of this arrangement is that the two eyes’ views need to be resolved to yield singular vision, which is normally accomplished by fusion or suppression of one of the eye’s inputs. This binocular processing has been shown to occur in cortex, subsequent to thalamic processing. Here, we show that neurons in the lateral geniculate nucleus (LGN) receiving excitatory retinal input from one eye can be suppressed by high-contrast visual stimulation of the other eye, indicating that the geniculate serves as a precortical site of binocular processing.

## Introduction

The lateral geniculate nucleus (LGN) is the main recipient of the outputs of the two eyes in primates. Its anatomic organization raises the question of the role it plays in resolving binocular inputs to support singular vision. In diurnal primates, the LGN comprises distinct layers and virtually all neurons within each layer receive direct inputs from only one eye (Kaas et al., 1972). Neurons within a layer receive input from one eye and neighboring layers contain neurons that receive input from the other eye. Congruent with their inputs, almost all LGN neurons are monocular, exclusively excited by stimulation of one eye. Nevertheless, LGN neurons might interact across layers (Campos-Ortega et al., 1968; Saini and Garey, 1981) or receive inputs from structures with binocular neurons (Hubel and Wiesel, 1968; Lund et al., 1975). The effects of these influences could manifest as a difference in magnitude of visual responses under binocular stimulation relative to monocular stimulation.

Studies in cats have well established that spike rates of most LGN neurons are altered by binocular stimulation (Sanderson et al., 1971; Schmielau and Singer, 1977; Xue et al., 1987; Tong et al., 1992; Sengpiel et al., 1995). Binocular modulation also occurs in the primate. Unlike in the cat, related work in macaques has not focused on visual contrast. Instead, previous work on binocular modulation either introduced luminance differences across the eyes (Rodieck and Dreher, 1979; Schroeder et al., 1990) or introduced stimuli at one contrast level (Marrocco and McClurkin, 1979). One reason to address this question in macaques is that cat LGN differs anatomically from primate LGN (for review, see Dougherty et al., 2019). Assessing binocular modulation in the LGN as a function of visual contrast in both eyes would reveal how interactions between the eyes are influenced by the strength of each eye’s signal. Contrast-dependent binocular interactions in the LGN could have implications for known psychophysical phenomena, such as interocular suppression.

The primate LGN is composed of three major cell classes, parvocellular (P), magnocellular (M), and koniocellular (K) neurons, with known functional and anatomic distinctions. Known differences in contrast sensitivity among these parallel pathways (Shapley et al., 1981; Derrington and Lennie, 1984; Norton et al., 1988; Lee et al., 1989a; Sclar et al., 1990) as well as their physical distribution could impact whether contrast-dependent binocular interactions occurs for these groups. Based on the studies that assessed general binocular modulation, it remains unclear whether this modulation occurs only for M or both P and M neurons (Marrocco and McClurkin, 1979; Rodieck and Dreher, 1979). Furthermore, whether K neurons, intercalated between different eye-dominant layers, show binocular modulation in macaques is an outstanding question. Recent work in anesthetized marmosets, focusing on K layers, found that about one third of K neurons get excitatory binocular input (Zeater et al., 2015), and that most K neurons with excitatory responses to high-contrast stimuli were suppressed by binocular stimulation (Belluccini et al., 2019). A second question is how binocular modulation of single neurons may be impacted by anesthesia, as anesthesia is known to affect binocular processing in this structure (Schroeder et al., 1988). Only one study thus far has considered LGN binocular processing in the awake state (Schroeder et al., 1990). However, these measures were based on population spiking, which could have included spikes from neighboring eye dominate layers.

We sought to determine how visual contrast viewed by both eyes impacts the visual response of LGN neurons receiving direct input from one eye only in awake primates. To do this, we varied the contrast of stimuli shown to each eye independently to adjust the signal strength each eye carries. Two macaques viewed drifting sine-wave gratings presented to one or both eyes while spiking of LGN neurons was recorded with a linear multicontact electrode array. We found that significant binocular modulation occurred when a high contrast stimulus drove the non-dominant eye while a low contrast stimulus was presented to the dominant eye. Individual LGN units showing this pattern comprised P, M, and K groups. This result suggests that inhibitory input from the non-dominant eye can suppress LGN neurons in awake primates only if the dominant eye input is too weak to overcome this inhibition. In other words, there is a weak, antagonistic relationship between the two eyes’ signals within primate LGN that only becomes evident at certain interocular contrast differences. This finding resolves earlier discrepant findings as to types of neurons showing binocular modulation, and suggests that the LGN is a precortical binocular processing site that may initiate the process of resolving discrepant contrast differences across the eyes.

## Materials and Methods

Two adult monkeys (*Macaca radiata*, one male) were used in this study. All procedures followed regulations by the Association for the Assessment and Accreditation of Laboratory Animal Care (AAALAC), the University’s Institutional Animal Care and Use Committee (IACUC) and National Institutes of Health (NIH) guidelines. Each monkey received nutritionally complete food biscuits, quantity based on recommendation by the veterinarian, in the morning and afternoon every day. In addition, animals were provided fresh produce and other forms of environmental enrichment on five or more days a week. The animals were water regulated during the weeks that the experiments were conducted. They were trained to fixate and received juice reward following completed trials.

### Surgical procedures

Before experiments began, each monkey underwent two separate surgeries conducted under sterile conditions. In the first surgery, the animals were implanted with a custom-designed plastic head holder. In the second surgery, a craniotomy was made and a plastic recording chamber (Crist Instruments) was implanted. The craniotomy was centered on stereotaxic coordinates above the LGN (anterior-posterior: 7 mm, medial-lateral: 12 mm). The head holder and the recording chamber were attached to the skull using either transcranial ceramic screws or titanium screws (Thomas Recording) and self-curing dental acrylic (Lang Dental Manufacturing). Animals were administered isoflurane anesthesia (1.5–2.0%), and vital signs, such as blood pressure, heart rate, SpO_2_, CO_2_, respiratory rate and body temperature were continuously monitored throughout both surgeries. Following surgery, each monkey was administered analgesics (buprenorphine) and antibiotics (ceftiofur sodium, unless otherwise directed by the veterinarian) by intramuscular injection. Researchers, facility veterinarians, and animal care staff closely observed the animals for at least 3 d following surgery.

### Visual display

**Figure 1.**
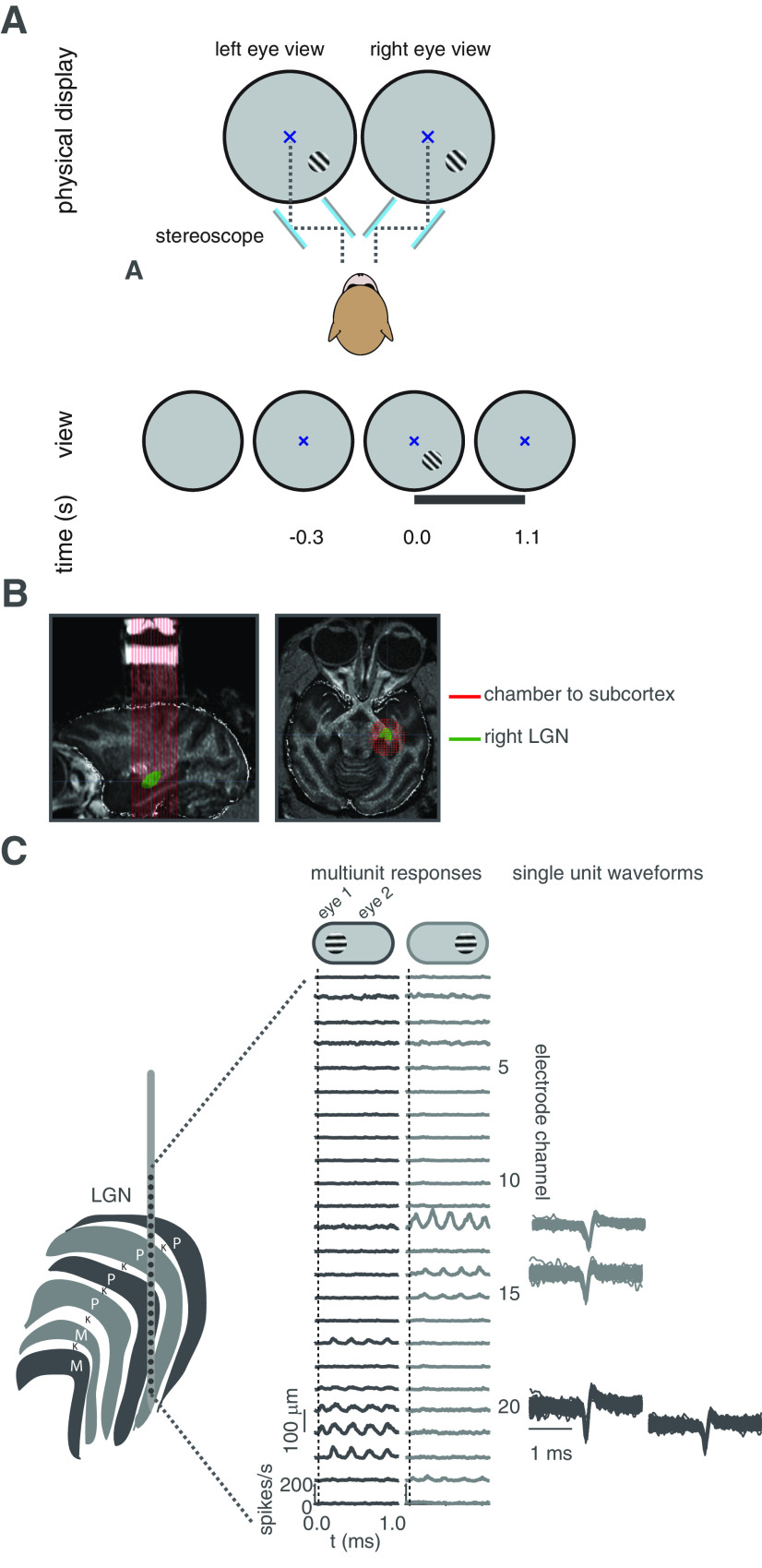
Experimental setup. ***A***, Visual stimuli were presented through a mirror stereoscope consisting of two pairs of mirrors angled so that the subjects fused the left and right sides of the display. Animals fixated a central cross that was presented dioptically before a monocularly or binocularly presented drifting sine-wave grating appeared over the receptive field of the recorded neurons for 1000–1100 ms. Schematic not shown to scale. ***B***, Sagittal (left) and axial (right) MRI of monkey I34 used to guide electrode placement within the well placed over the right hemisphere’s LGN. ***C***, To-scale schematic of linear multicontact electrode array in the LGN (left). In the example session shown, multiunit activity on nine contacts exhibited monocular, linear visual responses to the drifting grating, with eye dominance changing across presumed layer boundaries. Some, but not all, of these spikes were classified as single units (right). See Materials and Methods for details.

Stimuli were presented on a linearized cathode-ray tube monitor with a refresh rate of either 60 Hz with resolution 1280 × 1024 (for 1 unit) or 85 Hz with resolution 1024 × 768 (for 50 units), or on a linearized LED (VPixx) display with a refresh rate of 120 Hz with resolution 1920 × 1080 (for 15 units). Visual stimuli were generated using custom-written code for MonkeyLogic (Asaad et al., 2013) in MATLAB (R2012-2014; The MathWorks) on a PC operating Windows 7 or Windows 10 with an NVIDIA graphics card. The stimuli were viewed through a calibrated stereoscope consisting of infrared (IR)-light passing cold mirrors (Edmund Optics; Fig. 1*A*). The experimental setup was configured so that the animal’s right eye viewed stimuli presented on the right side of the monitor and the animal’s left eye viewed stimuli on the left side of the monitor. A black, non-reflective septum was placed between the monitor and the back side of the mirrors, effectively dividing the left and right sides of the apparatus, to prevent light scatter from one side of the monitor to the opposing eye.

IR light-sensitive cameras, placed directly behind the cold mirrors on the stereoscope, were used to track gaze position with commercially available eye tracking software (Eye Link II, SR Research). Both eyes were tracked using two separate cameras in the majority of recording sessions. Gaze position was converted to an analog signal and inputted to MonkeyLogic/MATLAB (NIDAQ PCI-6229) at 1 kHz. The stereoscope was calibrated to facilitate binocular fusion of the left and right sides of the monitor using a behavioral task that relied on acquiring the same gaze position for corresponding locations on each side of the monitor at the beginning of each recording session. In this task, the animal was required to move their gaze position to a single fixation cue that was placed at the center of the left side of the monitor, shown to the left eye only, and gaze position was recorded. The single fixation cue was moved to eight other locations one-at-a-time, spaced equidistant from the left monitor’s center up to 10°. Gaze position was recorded for each cue location. This procedure was repeated for the right side of the monitor. Then, gaze positions for corresponding cue locations from the left and right sides of the monitor were compared. Overlapping gaze positions for corresponding cue locations suggest the mirrors are optically aligned for binocular fusion. We confirmed that binocular fusion could occur by showing a central fixation cue to the left eye and right eye in alternation, monitoring for any change in eye position. An oval aperture or set of intersecting circles in each corner was displayed at the edge of each half-screen to further aid fusion.

Cone-isolating stimuli were generated by convolving the monitor (R, G, B) channel spectral power distribution with absorbance templates (Lamb, 1995) for cones with peak sensitivity in short-wavelength (S; 420 nm), medium-wavelength (M; 530 nm), or long-wavelength (L; 558 nm) bands of the visible spectrum. Monitor spectral power distributions were measured using a PR-655 photometer (Photograph Research). Cone templates were adjusted to account for lens absorption and pigment self-screening assuming axial absorbance of 0.15 and outer segment length 20 nm. No correction for macular pigment was incorporated because the vast majority of receptive field eccentricities were above 2°.

### Neurophysiological recordings and data acquisition

Following the second surgery, the location of the LGN was mapped by sampling different locations of a grid with 1 mm spacing (Crist Instruments) that was placed inside the chamber (Fig. 1*B*). Specifically, a linear multicontact array (V-Probe, U-Probe, Plexon Inc.) with either 24 or 32 contacts of 0.1-mm intercontact spacing was lowered through a guide tube placed within the chosen grid hole. After the probe tip left the guide tube, it was moved at rate of ∼10 μm/s to reach the LGN. Each encountered receptive field was first manually mapped using drifting sine-wave gratings, Gabor-filtered sine wave gratings or rectangular bars of high contrast and luminance. These stimuli were moved on the display while the animal fixated a central cue. The stimulus was then placed in corresponding RF locations in both eyes to verify that excitatory responses were monocularly driven. The geometry of the LGN and angle of approach meant that sometimes spiking on several electrode contacts could be stimulated by the same stimulus (∼2.5–5° diameter), but this was not always the case (Fig. 1*C*). In all, 29 sessions of LGN data were recorded with a linear multicontact array, and 11 sessions were recorded with a standard single-contact tungsten microelectrode (FHC Inc).

During each session, extracellular voltage fluctuations (filtered at 0.5 Hz to 30 kHz) were recorded inside an electromagnetic-shielded booth. These signals were amplified, filtered and digitized using a 128-channel Cerebus Neural Signal Processing System (NSP; Blackrock Microsystems). A bandpass filtered (0.5 Hz to 7.5 kHz) signal sampled at 30 kHz was saved for offline analysis. The NSP system also digitized the analog output of an IR-based eye tracking system (EyeLink II, SR Research) and the output of a photodiode (OSI Optoelectronics) that was placed on the monitor to timestamp stimulus-related events. The NSP also recorded digital event codes that were sent from the behavioral control system (MonkeyLogic; Asaad et al., 2013). The photodiode signal and event markers were used to align the neural data with visual and behavioral events.

All neurophysiological signals were extracted offline using custom written code in MATLAB (2016a and 2019a; The MathWorks). Single neurons were extracted with KiloSort (Pachitariu et al., 2016), using default parameters for sorting and cluster merging, with a few exceptions such as changing the threshold to detect spikes to 2.5 SDs. For all multicontact electrode sessions, the results of KiloSort were viewed in Phy, an open source analysis package (https://github.com/cortex-lab/phy). Using Phy, clusters were manually split or merged when appropriate, and were rated for isolation quality. The channel on the probe on which the cluster was recorded was determined using code from the University College London Cortex Lab (https://github.com/cortex-lab/spikes).

Spike rates were downsampled to 1 kHz. For each neuron, spike times were converted to a time-varying signal using 0 to represent time points without a spike and 1 for time points where a spike was detected. The time-varying signal was then convolved using a Poisson distribution resembling a postsynaptic potential (Sayer et al., 1990), with the spike rate (R) computed at time (t):

$$R\left(t\right)=\left[1-\text{exp}(-\frac{t}{\tau_{g}})\right]\text{*}\left[\text{exp}(-\frac{t}{\tau_{d}})\right],$$

where $\tau_{g}$ and $\tau_{d}$ are the time constants for growth and decay, respectively. Values of 1 and 20 for $\tau_{g}$ and $\tau_{d}$ respectively were used based on previous studies (Hanes et al., 1995). After convolution, units were converted to spikes per second by multiplying the signal by the sampling frequency.

### Experimental design and statistical analysis

During each session, data were collected while the animal viewed drifting sine-wave gratings of varying contrast presented to one or both eyes (this paradigm is hereinafter referred to as the binocular contrast paradigm). The animal was required to fixate within a 1–2° radius around the central fixation cue (0.5°). All gratings were presented at temporal frequency of 4 Hz (except for one session at 8 Hz) and spatial frequency of 1 cycle/°. Within each recording epoch, gratings were presented at constant orientation (*N* = 27 units horizontal, *N* = 19 vertical, *N* = 10 non-cardinal).

In addition to the binocular contrast paradigm, we collected data to identify whether neurons belonged to the P, M, or K subclass of LGN neurons. For 27 sessions, we collected spiking data while we presented cone-isolating stimuli designed to modulate along the L^+^M^+^/L^–^M^–^, L^+^M^–^/L^–^M^+^, or S^+^/S^–^ axes in DKL color space (Derrington et al., 1984; Lee et al., 1989a). In each trial, the animal fixated while a cone-isolating stimulus with temporal frequency of 4 Hz and spatial frequency of 1 cycle degree^−1^ drifted over the RF location of either the dominant eye, or both eyes simultaneously for ∼1000–1100 ms. For 12 other sessions, we collected spiking data while we presented alternating (red-to-green or blue-to-yellow) color patches to the receptive field while the animal fixated. We used converging evidence, including the shape of the contrast response curves, the responses to the spectral stimuli, and contextual information from multicontact arrays (chiefly, recording depth and the character of background multiunit activity) to label each unit as part of the P, M, or K functional streams (Wiesel and Hubel, 1966; Shapley et al., 1981; Derrington et al., 1984; Derrington and Lennie, 1984; Norton et al., 1988; Lee et al., 1989a; Sclar et al., 1990; White et al., 1998, 2001). If the overall spike density function across a single file with the binocular contrast paradigm had a mean of <1.9 spikes/s, we excluded the cluster from further analysis. We also excluded units that had ambiguous or atypical contrast response functions (CRFs).

To assess the stability of the units during the binocular contrast paradigm, we summed the spike counts of the time-varying, trial-aligned spiking data for the period 600 ms before stimulus onset through 1300 ms following stimulus onset. We computed a running average (*movmean.m* from MATLAB, with window length equal to 20 ms) of these counts across trials, then identified likely points of instability in the mean spike rate (*findchangepts.m* from MATLAB). We removed periods of instability using this algorithm. We checked the results of the algorithm for each unit. In rare cases, we manually removed periods of instability based on judicious visual inspection.

Neurons were further analyzed whether they were recorded for at least 12 trials for monocular and binocular conditions, each with 0.5 contrast or higher. For each unit and for every trial, we computed the autocorrelation on the time-varying, trial-aligned spiking data for the period between 0 and 1100 ms relative to stimulus onset. We normalized the autocorrelation for each trial by dividing by the total number of spikes in that trial. We then calculated the power spectral density (PSD) of the autocorrelations using a method designed to extract oscillatory components from 1/f-trending data (Wen and Liu, 2016). We refer to the PSD for the oscillatory (rhythmic) components as the oscillatory PSD, and for the (arrhythmic) 1/f components as the fractal PSD. Recorded units were considered to show significant visual response if two requirements were met: (1) power of the oscillatory PSD at the grating drift frequency exceeded the power of the fractal PSD at that same frequency for blank (no stimulus) trials based on a one-tailed *t* test (α = 0.05); and (2) the power of the oscillatory PSD at the grating drift frequency exceeded the power of the fractal PSD at the same frequency based on a one-tailed *t* test (α = 0.05).

Responses to visual stimuli were based on the power of the oscillatory PSD at the grating drift frequency. Binocular modulation was assessed by the percent difference between the monocular response and the binocular response for each unit. Outliers (values that were more than three scaled median absolute deviations from the median) were excluded from both the figures and statistical analyses. Distributions of binocular modulation were fit with a non-parametric kernel smoothing function, with a normal distribution shape for the component curves, in MATLAB.

Statistical significance of individual units was estimated using receiver operating characteristic (ROC) analysis, comparing binocular responses to the monocular responses for each unit (*perfcurve.m* in MATLAB). Specifically, we used the power at the drift rate of the stimulus based on the oscillatory PSD described above, or the F1 response. To assess significance, we ran a random (Monte Carlo) shuffle control in which we shuffled, with replacement, across both conditions, creating two surrogate conditions. We then calculated the ROC curve for these surrogate comparisons. Because we tested for both binocular suppression and binocular facilitation, we used a threshold quantile of 0.975 for α = 0.05. Specifically, if the area under the curve exceeded the 0.975 quantile of the shuffled distribution of AUC values, the unit was considered to show significant binocular modulation for that condition. One exception to this criterion was made and explictly noted, where units showing suppression at α = 0.2 (or AUC > 0.9 of the shuffled distribution) were included to increase *N*. To determine the direction of the modulation (binocular suppression or facilitation), we compared the means of the binocular and monocular conditions. If the mean response was greater under binocular stimulation than monocular stimulation, we deemed the neuron to show binocular facilitation; under the converse condition we deemed the neuron to show binocular suppression. The contrast levels used across recording days was not perfectly consistent. To consolidate data for population averages, we grouped the following contrast ranges: 0.0, 0.025–0.05, 0.106–0.135, 0.318–0.368, 0.8 –1.0. These ranges were based on clustering of the contrast levels sampled across units. For visualization, data are presented at the median value of the relevant range.

### MRI

MRI was used to aid electrode placement. Animals were anesthetized using the procedure outlined above then placed in a Philips Achieva 3T MRI scanner (80 mT/m gradient strength, 200T/m/s slew-rate; Koninklijke Philips N.V.) Vital signs were monitored continuously. T1-weighted 3D MPRAGE scans were acquired with a 32-channel head coil equipped for SENSE imaging. Images were acquired using a 0.5-mm isotropic voxel resolution with the following parameters: repetition time (TR) 5 s, echo time (TE) 2.5 ms, flip angle 7°.

### Code accessibility

Custom-written code used for this study is available from the corresponding author on request.

## Results

Our primary goal was to determine how visual responses of LGN neurons in awake primates are influenced by the eye from which they do not receive direct retinal input (the non-dominant eye). In common with prior work in anesthetized animals (Tong et al., 1992; Sengpiel et al., 1995; Belluccini et al., 2019), we tested this question by varying the contrast of the stimulus presented to each eye, thereby adjusting the relative strength of the signal each eye carried. In the following sections, we first consider whether the presence of stimulus in the non-dominant eye impacts visual response to stimuli presented at different contrasts to the dominant eye. Then, we consider how binocular modulation is impacted by the contrast in the non-dominant eye.

One important feature of our experimental setup was that all stimuli were presented through a calibrated mirror stereoscope (Fig. 1; Materials and Methods). Thus, we were able to hold mean luminance constant across both eyes and avoided effects of short-term monocular deprivation that can arise if one eye is patched (Lunghi et al., 2015; Zhou et al., 2015; Binda et al., 2018; Wang et al., 2020). Under monocular conditions, the dominant eye of the recorded neurons was stimulated at either low (0.12), medium (0.34), or high (0.9) contrast levels. In pseudo-randomized separate trials, the dominant eye viewed the same (low, medium, or high contrast) stimulus while the non-dominant eye was presented with either a high (0.9) or medium (0.34) contrast stimulus. In total we collected spiking data from 56 LGN single units (41 units in monkey I34, 15 in monkey B52) across 40 sessions (27 sessions in I34, 13 in monkey B52). Note that while we used multicontact arrays, we were unable to record from multiple units simultaneously for most sessions. The reason for this limitation is that we entered the brain orthogonal to the cortex above the LGN, and because of the shape of the LGN, the receptive field locations across the probe did not typically align. The benefit of the multicontact array was that the multiunit hash on the contacts provided context of the location of the electrode in the brain.

**Figure 2.**
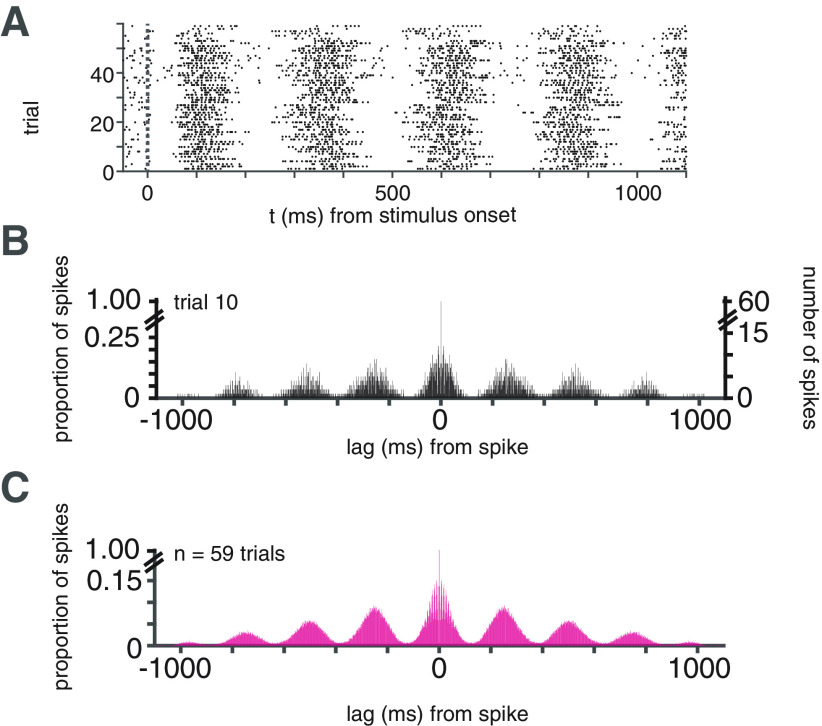
Schematic of autocorrelation-based analysis of LGN linear visual responses. ***A***, Note small changes in the onset of responses across trials likely because of small shifts in the animals’ fixation position (see Materials and Methods). Calculation of autocorrelation of the spike train during the visual stimulation period for example trial. ***B***, Conversion of autocorrelation into units of spiking probability as function of lag for single trial. ***C***, Average spiking probabilities across trials.

Macaques tend to make fixational eye movements such as microsaccades as well as small shifts in initial fixation position from trial-to-trial. In this study, variability in initial fixation position from trial-to-trial was substantial enough to presumably cause LGN receptive fields to shift in spatial position with respect to the stimulus. This variance in initial fixation position, and therefore initial phase of the grating stimulus over the RF, could cause the LGN spiking responses exhibit eye movement-related shifts in phase, both across and within trials (Fig. 2*A*). For example, the range of the initial (mean position between 0 and 15 ms poststimulus onset) horizontal and vertical eye positions was 0.99° and 1.4°, respectively, for a file from the session shown in Figure 2*A*. We obviated this problem by calculating the autocorrelation of spikes for every trial (Fig. 2*B*), followed by averaging to yield the phase-aligned linear response to the stimulus (a grating drifting at 4 Hz; Fig. 2*C*).

**Figure 3.**
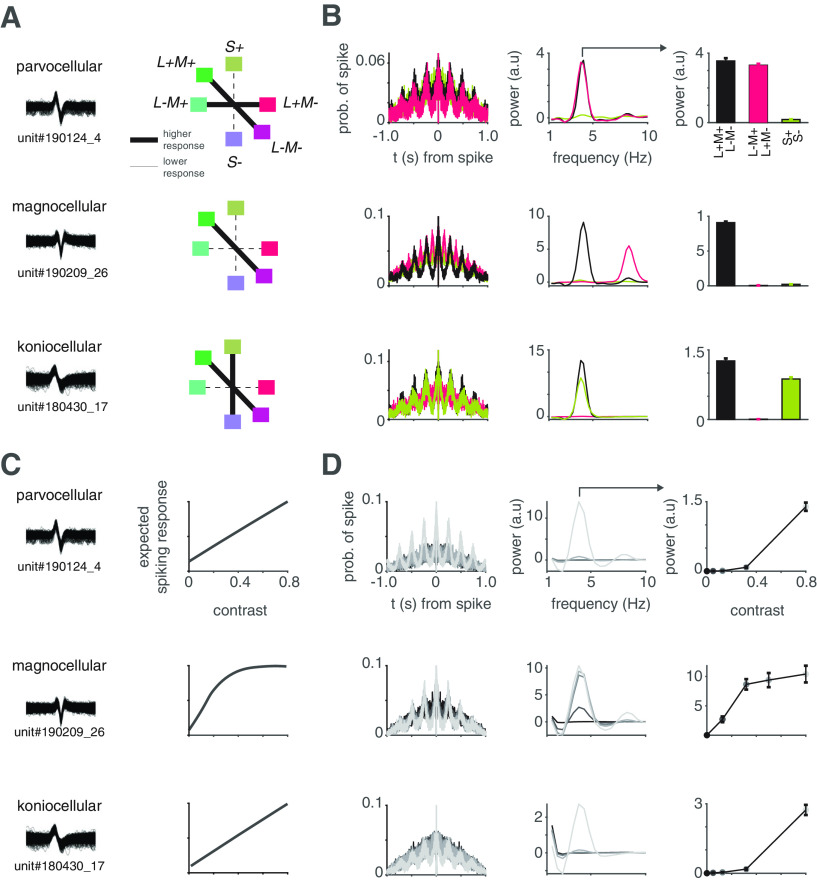
Neurophysiological characterization of P, M, and K LGN neurons. ***A***, Waveforms from individual spikes (*N* > 1000) for representative P, M, and K units (left). Expected responses across L^+^M^+^/L^–^M^–^, L^−^M^+^/L^+^M^–^, and S^+^/S^–^ stimulus conditions, expressed in DKL color space for each neuron type (bolder lines indicate stronger responses; right). ***B***, Average spike autocorrelation for L^+^M^+^/L^–^M^–^, L^−^M^+^/L^+^M^–^, and S^+^/S^–^ stimulus conditions (left), average PSD on the autocorrelations (center), and the mean power of the PSD at the temporal frequency of the drifting grating (right) for each example P (top), M (middle), and K unit (bottom). Error bars indicate SEM. Power is the difference between the power of the signal and the estimated fractal power (see Materials and Methods). Note that the prominent 8-Hz modulation for the 1 cycle/° 4-Hz drifting L^+^M^–^/L^–^M^+^ stimulus results from M neurons receiving input from retinal ganglion cells that sum inputs from L and M cones (see Lee et al., 1989b). ***C***, The same units as in ***A*** with expected contrast response curve shapes based on published data cited in Results. ***D***, Same as in ***B*** but for different contrast levels, with each line representing a different contrast level, where darker colors represent lower contrast levels. Data within a range around each contrast point were averaged (see Materials and Methods).

**Figure 4.**
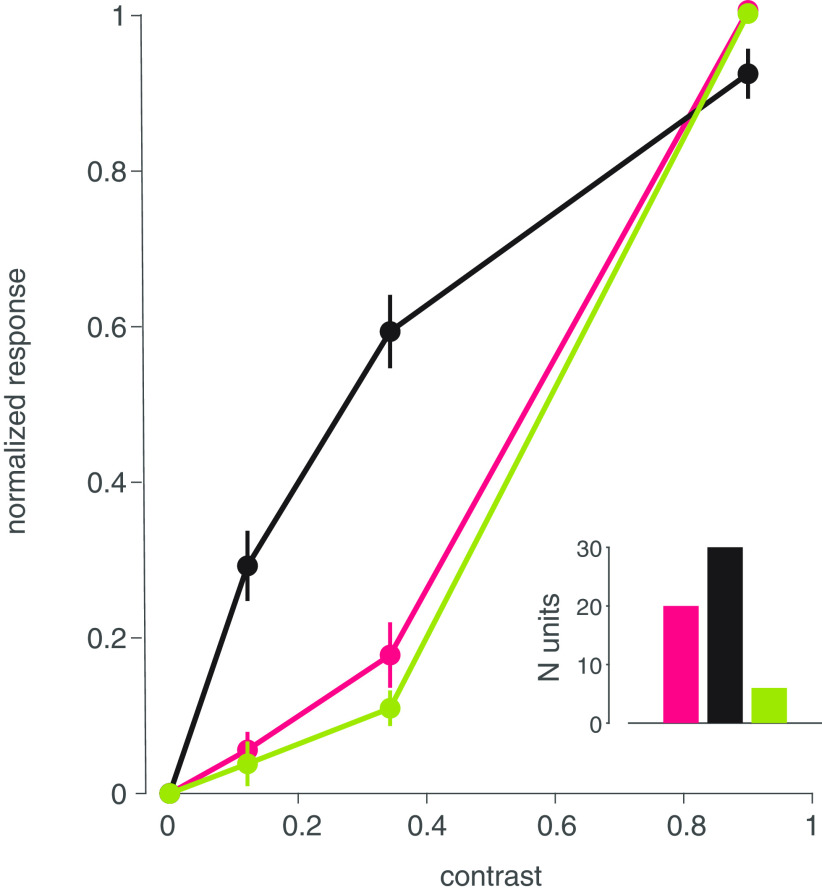
Mean CRFs for putative P, M, and K neurons. Responses at different contrast levels were normalized between 0 and 1 for each unit, and averaged across units belonging to each group, with the maximum number of units in each group shown above. Error bars indicate SEM. Data within a contrast range around each contrast point listed were averaged (see Materials and Methods).

We first identified each LGN neuron as P, M, or K by analyzing their respective responses to stimuli of varying spectral content and contrast (see Materials and Methods; Lee et al., 1989b). Responses to different spectral (cone-isolating) stimuli are shown for example P, M and K units in Figure 3. In all, we identified 20 P, 30 M, and 6 K neurons based on their spectral responses (see Materials and Methods). The average CRFs of each of these groups matched expectations based on previous reports (Fig. 4; Shapley et al., 1981; Derrington and Lennie, 1984; Norton et al., 1988; White et al., 2001).

### Binocular modulation as a function of contrast in the dominant eye

**Figure 5.**
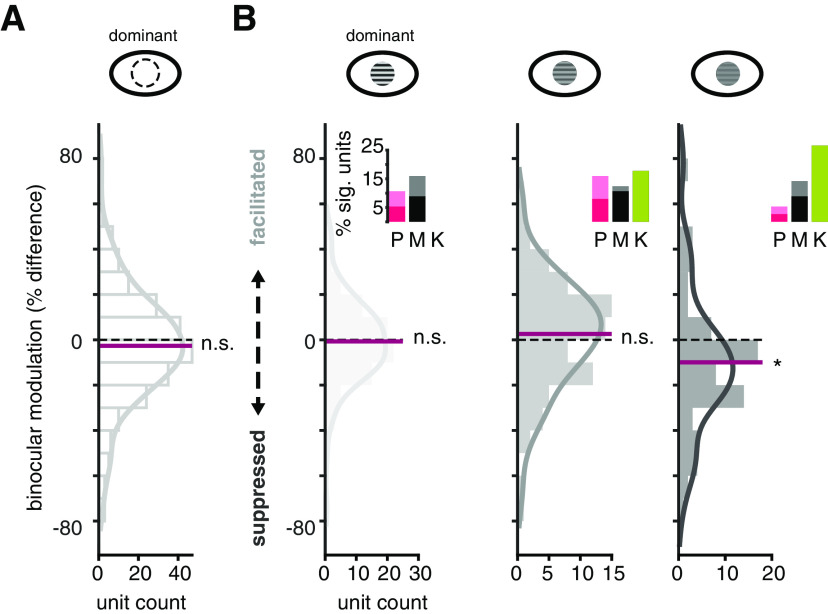
Binocular modulation occurs with low contrast in the dominant eye. ***A***, Visual response difference between monocular and binocular stimulation across all contrast levels for all units. Percent difference calculated between monocular and binocular stimulation, with contrast matched in the dominant eye, shows no effect of stimulating the non-dominant eye. Black, horizontal dashed line demarcates division between binocular facilitation and suppression. Maroon, horizontal line marks the median of the distribution. Solid gray represents a non-parametric, kernel smoothing fit to the data. N.S. indicates binocular modulation across sample is not significantly different from 0, alpha = 0.05. ***B***, Visual response difference between monocular and binocular stimulation for high (0.9), medium (0.34), and low (0.12) contrast gratings shown to the dominant eye, from left to right respectively. Bar graph insets show proportion of significant units (ROC analysis, α = 0.05) for each P, M, and K samples at respective contrast levels. Colors as in Figure 4. Light shading of the bars corresponds to facilitated units. Asterisk indicates sample shows binocular modulation is significantly different from 0, alpha = 0.05.

We first considered whether there was a difference between binocular stimulation and monocular stimulation of LGN neurons in general. To test for binocular modulation across the entire sample, we calculated the percent difference between monocular responses and binocular responses, with the same contrast in the dominant eye and contrast above 0 in the non-dominant eye. The median percent difference between monocular and binocular conditions was −2.65%, with no significant difference from 0 (Wilcoxon signed-rank, *p* = 0.11) across the distribution. Therefore, there was no effect of binocular stimulation if contrast levels were not considered (Fig. 5*A*).

Next, we evaluated whether binocular modulation depends on contrast shown to the dominant eye. When we considered stimulation of the dominant eye at high, medium, and low contrast levels, a clear pattern emerged: non-dominant eye effects were present when the dominant eye was stimulated at low contrast (median: −9.95%; Wilcoxon sign-rank, *p* = 0.0024; Fig. 5*B*) but not at medium (median: 2.58%; Wilcoxon sign-rank, *p* = 0.35) or high (median: −0.73%; Wilcoxon sign-rank, *p* = 0.77) contrast levels. Based on the distribution and median of the sample, binocular modulation was predominantly suppressive. Therefore, stimulating the non-dominant eye tended to reduce spiking responses of LGN neurons when the dominant eye was stimulated at low contrast.

We next considered whether binocular modulation was specific to one or more LGN subclasses. To do so, we assessed whether there was a significant difference between monocular and binocular stimulation for each unit in our sample that we had identified as P, M, or K as described above. We found significant binocular modulation in units belonging to all three (P, M, and K) subclasses (Fig. 5*B*). This analysis also revealed more instances of binocular suppression than facilitation at every contrast level in the dominant eye. There was no clear division across subclasses with respect to binocular modulation, except that none of the sampled K units showed significant modulation when high contrast was delivered through the dominant eye. These findings suggest that weak stimulation of the dominant eye allows for, predominantly suppressive, influences of the non-dominant eye across all major types of LGN neurons. This result motivated us to investigate more closely the effect of the non-dominant eye.

### Influence of the non-dominant eye

**Figure 6.**
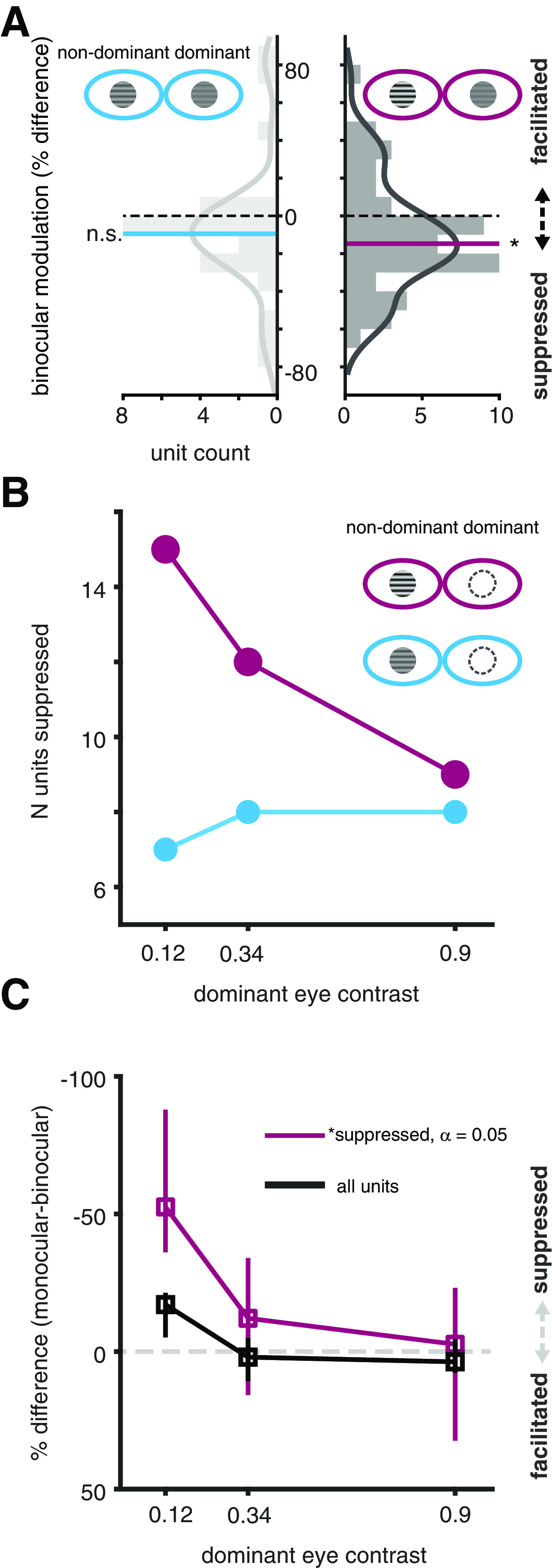
Comparison of binocular modulation at two different non-dominant eye contrast levels. ***A***, left, Visual response difference between monocular and binocular stimulation with low (0.12) contrast in the dominant eye and medium (0.34) contrast in the non-dominant eye. Blue line marks the median of the distribution. Right, Same but with high (0.9) contrast in the non-dominant eye. Maroon line marks the median of the distribution. Other conventions as in Figure 5. ***B***, Number of units that show suppression (ROC analysis, α = 0.2) across dominant eye contrast levels with medium (blue) and high (maroon) contrast in the non-dominant eye. ***C***, Median binocular modulation as a function of contrast in the dominant eye for units showing significant modulation (maroon, ROC, α = 0.05, *N* = 6) with high interocular contrast imbalance (low contrast dominant eye, high contrast non-dominant eye), as well as across the whole sample (black line). Error bars are 95% confidence intervals on the medians.

As a next step, we compared effects of a medium and high contrast stimulus delivered through the non-dominant eye. Doing so, we found that significant suppression occurred only when we presented a high contrast stimulus in the non-dominant eye and a low contrast stimulus in the dominant eye, though a medium contrast stimulus in the non-dominant eye trended toward significance (low contrast: Wilcoxon sign-rank, *p* = 0.013; medium contrast: Wilcoxon sign-rank, *p* = 0.058; Fig. 6*A*). In other words, significant suppression across the sample was limited to instances where the suppressive drive from the non-dominant eye was strong and the excitatory drive from the dominant eye was low (and thus perhaps insufficient to override the suppressive influence).

If the non-dominant eye influences LGN responses of the dominant eye in a contrast-dependent fashion, we would expect a trend with respect to the fraction of LGN units that show significant suppression across the contrast range. We thus considered the percentage of units showing binocular suppression (ROC analysis, α = 0.2) as a function of contrast in the dominant eye (Fig. 6*B*). Using a high contrast stimulus in the non-dominant eye, we found a negative slope (linear regression, slope = −7.26, *p* = 0.16, df = 1) with the most units showing suppression at low contrasts and fewest at the highest contrast. There was no clear trend with medium contrast in the non-dominant eye (linear regression, slope = 1.04, *p* = 0.49, df = 1). One interpretation of this flat line is that it represents a noise floor, as in, weak stimulation of the non-dominant eye has no measurable impact.

Another way to assess whether the non-dominant eye influences responses of the dominant eye in a contrast-dependent fashion is to study the binocular modulation of units showing significant suppression at low contrast in the dominant eye (and high contrast in the non-dominant eye). While there were only 6 units that showed significant modulation at this level, their median binocular modulation decreased gradually with increasing contrast in the dominant eye (Fig. 6*C*, purple line). Assessing binocular modulation with a high contrast stimulus in the non-dominant eye across all units, there was a similar trend (Fig. 6*C*, black line). However, this trend did not reach significance after Bonferroni correction (low vs medium: Wilcoxon sign-rank, *p* = 0.067; medium vs high: Wilcoxon sign-rank, *p* = 0.86; low vs high: Wilcoxon sign-rank, *p* = 0.032).

## Discussion

As far as we are aware, this is the first study to investigate contrast-dependent binocular interactions in the LGN of awake macaques. The main finding from this study is that significant binocular suppression occurs across all major LGN cell classes when the non-dominant eye is stimulated at high contrast and the dominant eye is stimulated at low contrast. The predominant effect of this kind of imbalanced binocular stimulation was suppression of LGN visual responses. This result is congruent with previous work, and reminiscent of findings from psychophysical and modeling studies that suggest an antagonistic relationship between the two eyes at a monocular stage of visual processing (Blake, 1989; Ding and Sperling, 2006; Baker et al., 2007; Ding and Levi, 2014).

### Relationship to prior work

One prior study of LGN units in anesthetized macaques reported binocular interactions for both P (X) and M (Y) neurons (K neurons were not distinguished; Marrocco and McClurkin, 1979). A different study tested for changes from baseline firing when a stimulus was presented to the non-dominant eye alone (non-dominant suppression) and reported binocular modulation only for M (Y) neurons (Rodieck and Dreher, 1979). We did not test stimulation of the non-dominant eye alone in the present study. Our results agree with Marrocco and McClurkin (1979) in that we observed binocular modulation in both P and M neurons.

Binocularly-driven excitatory responses have been reported for ∼30% of K neurons in marmoset monkeys (Cheong et al., 2013; Zeater et al., 2015). In a more recent study, Belluccini et al. (2019) observed that most marmoset K neurons with excitatory responses to high-contrast stimuli showed binocular suppression. Consistent with this result, we observed only binocular suppression at low and medium contrasts in our relatively small sample of macaque K neurons.

The findings reported here are also in accordance with findings of binocular suppression and facilitation of LGN multiunit activity in awake macaques (Schroeder et al., 1990). These authors report ∼35–100% of sites show binocular suppression, with exact percentage depending on layer, except in layer P4 where no binocular suppression was observed. In the present study, the highest proportion of total neurons that showed binocular suppression was ∼25%, which was observed using low contrast in the dominant eye. However, given the difference in underlying signals (population spiking vs single unit activity), it is difficult to compare these measures directly. Another consideration is that we compared monocular and binocular stimulation with drifting gratings of constant luminance that covered the RF, whereas Schroeder et al. (1990) used a high-intensity flash (strobe) stimulus.

In summary, this study and extant literature converge to the conclusion that binocular interactions in LGN is primarily suppressive in nature. This conclusion extends across anesthetized macaques and marmosets (Marrocco and McClurkin, 1979; Rodieck and Dreher, 1979; Belluccini et al., 2019) as well as cats (Sanderson et al., 1971; Schmielau and Singer, 1977; Rodieck and Dreher, 1979; Xue et al., 1987; Tong et al., 1992; Sengpiel et al., 1995) and to awake macaques (Schroeder et al., 1990; present study).

### Antagonistic relationship between the two eyes

Across the sample, significant binocular modulation occurred only when there was a high contrast stimulus in the non-dominant eye and low contrast stimulus in the dominant eye. This result suggests that LGN neurons in awake primates are inhibited by the eye from which they receive no direct retinal input when that eye carries a signal much stronger than the eye from which they receive direct retinal input. Given that most of this modulation was suppressive, the pattern observed suggests a competitive relationship between the two eyes’ signals at the earliest level of non-retinal visual processing.

The pattern of binocular modulation observed here is consistent with the phenomenon of interocular suppression more generally, in that more suppression occurs with greater contrast differences between the eyes (albeit only when that difference favors the non-dominant eye). The result reported in this study is also congruent with previous work in the cat that suggested interocular suppression occurs in about half of cat LGN neurons (Sengpiel et al., 1995).

Interocular suppression has been suggested to play a major role in binocular rivalry, the visual phenomenon resembling alternating views of each eye’s perspective that arises when the images in the two eyes are too different to fuse (Blake, 1989). Note that in this study, we were exclusively focused on conditions that are known to induce binocular fusion: gratings of identical spatial position, orientation, spatial frequency and phase that only differed in interocular contrast. Interocular contrast differences alone are insufficient to induce binocular rivalry (Kertesz and Jones, 1970; O'Shea and Crassini, 1982; Ding and Sperling, 2006; Huang et al., 2010; Ding and Levi, 2017). Prior work in macaques suggest that LGN neurons do not alter their responses to rivalrous stimuli (Lehky and Maunsell, 1996), yet several human fMRI studies suggest otherwise (Haynes et al., 2005; Wunderlich et al., 2005). It thus would be interesting to revisit the role of different types of LGN neurons for binocular rivalry in future studies that incorporate perceptual report from the animal (Leopold et al., 2003).

The results presented here also call to mind models in which the two eyes share a mutually antagonistic, or competitive, relationship (Blake, 1989; Ding and Sperling, 2006; Baker et al., 2007; Ding and Levi, 2014): if the eyes compete at a monocular stage of processing, binocular interactions will be more likely to occur when disparate levels of contrast are presented to the two eyes. The non-dominant eye can reduce dominant eye responses to low contrast only if the contrast presented to the non-dominant eye is stronger than the contrast presented to the dominant eye. On the other hand, the neuron is less susceptible to the influence of the non-dominant eye if the contrast delivered through both eyes is more or less equal. Several models on binocular combination include interocular gain control at a monocular stage, before the two monocular streams are combined to a binocular signal (Truchard et al., 2000; Ding and Sperling, 2006; Meese et al., 2006; Baker et al., 2007). Indeed, the binocular suppression in the LGN we report in this study (see Figs. 5*B*, 6) qualitatively agrees with predictions for interocular gain control at a monocular stage. Note, however, that binocular suppression was statistically significant for the most extreme interocular contrast discrepancy only. While these results shed light on the functional architecture underlying how the two eyes interact at this early stage of visual processing, it is important to acknowledge the much more extensive binocular interactions that occur under broad stimulus conditions in primary visual cortex (Hubel and Wiesel, 1968; Smith et al., 1997; Moradi and Heeger, 2009). Thus, an alternative, albeit not mutually exclusive, explanation for binocular suppression in the LGN is that the suppression comes about through extraretinal circuits that mediate gain control in the LGN. For example, LGN contrast gain control has been shown to be impacted by cooling of visual cortical neurons (Przybyszewski et al., 2000). Gain control mechanisms in these or other binocular circuits could impact responses in LGN neurons through feedback projections.

As mentioned above, binocular suppression in the LGN was statistically significant only for the most extreme interocular contrast discrepancy, with higher contrast in the non-dominant eye. In individuals with normal binocular vision, contrast is often matched across the eyes. However, large interocular contrast differences might occur when one eye is closed, when one eye’s view is obstructed (e.g., by the nose) or when the eyes are misaligned. Indeed, in individuals with strabismic amblyopia, mismatched contrasts across the eyes is necessary for equal performance on psychophysical tasks because of interocular suppression (Li et al., 2011). In any case, imbalanced contrast conditions created in the laboratory are informative in that they shed light on the nature of the (antagonistic) relationship between the two eyes at this early stage of visual processing.

### Neurophysiological mechanism for binocular modulation in the LGN

Binocular modulation of LGN neurons in primates could arise, among other possibilities, through (1) local connections within the LGN, (2) connections between the TRN and LGN, or (3) connections from V1 to the LGN directly or via the TRN (for review, see Dougherty et al., 2019). Indeed, the source of binocular LGN modulation may be different for P, M, or K neurons, given the known specificity of corticogeniculate projections to the LGN (Briggs and Usrey, 2009). For K neurons specifically, binocular modulation could arise through connections between the superior colliculus and the LGN (Stepniewska et al., 1999; Zeater et al., 2019). Because of the variance in initial fixation position around the fixation cross (that presumably led to RF shifts with respect to the sinusoidal grating), we were unable to analyze the onset latency of modulation to infer the source of the modulation to distinguish between these possibilities. More work will be needed to determine the source of binocular modulation in the LGN.

In summary, the findings presented in this study build on previous experiments (Marrocco and McClurkin, 1979; Rodieck and Dreher, 1979; Schroeder et al., 1990; Belluccini et al., 2019) by showing that a minority of LGN neurons are sensitive to both eyes in awake primates. The pattern of modulation across contrast levels nevertheless suggests a role of LGN neurons in suppressing large interocular contrast differences.

## Synthesis

Reviewing Editor: Siegrid Löwel, University of Goettingen Decisions are customarily a result of the Reviewing Editor and the peer reviewers coming together and discussing their recommendations until a consensus is reached. When revisions are invited, a fact-based synthesis statement explaining their decision and outlining what is needed to prepare a revision will be listed below. The following reviewer(s) agreed to reveal their identity: Sergio Neuenschwander.

Results from the present study demonstrate that monocular LGN cells can be influenced by visual stimulation to the non-dominant eye. While effects are small, they provide insight into intrathalamic circuitry. In contrast to previous studies showing MUA data and recordings in anesthetized cats and monkeys, this study uses single cell recordings from the LGN of awake monkeys. Another strong point is that calibrated stimuli (same luminance across the eyes) were used.

The main goal of this study was to characterize the influence of contrast in binocular interactions. For this, moving gratings were used as stimuli (optimal spatial and temporal frequencies, three orientations). The study provides evidence of binocular suppression for the three functional pathways (P, M, and K-channels), specifically when the non-dominant-eye was stimulated at high contrast and the dominant-eye was stimulated at low contrast. While effects are modest and not evident for many cells, the results are compelling and not restricted to cells belonging to a particular parallel pathway. Statistical evaluation was sophisticated, based on receiver operating characteristic analysis. In general, the manuscript is well written and clear; the figures are clean and direct. The recording electrode positions were controlled using magnetic resonance imaging. Overall this is a high-quality paper.

Nevertheless, we feel that the manuscript needs some clarifications, and would benefit from some additional topics for discussion, as detailed below.

Major points

1. This study assumes that the experimental viewing conditions allowed for singular vision (the stimuli presented dichoptically were fused). But do we have evidence that this actually happened? I have the impression that the authors avoided the problem of rivalry, even though recognizing that the suppression was more significant when the contrast discrepancies between the two eyes were high. In this context, it would be most interesting if the authors could show data for grating stimuli differing both in contrast and orientation. In a condition of rivalry, are the contrast effects the same? This question is particularly relevant considering that the binocular interactions in the LGN are possibly mediated by layer VI binocular cell inputs from the cortex.

2. It seems that the authors gave a lot of importance to fixational eye movements as a confounding factor in their experiments (lines 305, 456). It was difficult to follow this argument in Figure 2A. It would be useful to see the eye traces juxtaposed to the responses in Figure 2A to examine the magnitude of these effects. How can 1 to 2 micro-saccadic events (1-second stimulus duration) falsify response latency estimation (line 456)?

3. We tend to think that the total size of the analyzed data (N= 56 units) is relatively small. This is true, especially if we consider that one of the main objectives of this study was a differential analysis across cell types (M, P, K). From a single monkey, only 15 cells were taken (across 13 recording sessions). In total, only six cells of type K were analyzed. Are these samples not too small given that linear multi-electrode matrices with 24 or 32 contacts were used for the recordings?

4. In the Discussion, line 445: Is interocular suppression used as a gain control mechanism across eye inputs? If these effects are seen in the LGN, why the authors think that they arise in the visual cortex? Please clarify this point.

5. Line 434. In this paragraph, the authors discuss their results in terms of competition between the two eyes, which is a reasonable topic for discussion. Another interpretation of the results that the authors should consider is whether they have identified a means to reveal details about the circuits that mediate gain control. LGN neurons are known to have contrast gain control that is augmented relative to their retinal inputs, indicating the involvement of extraretinal mechanisms. Results from the current study could be interpreted as evidence in support of extraretinal mechanisms for gain control, as well as for the idea that extraretinal gain control circuits are binocular. These ideas could be included to broaden the implications of the study’s results in the Discussion.

6. While it is difficult to think of situations where a stimulus in a specific region of space would have different contrasts for the two eyes, it could occur if the eyes were not aligned or if one eye is closed. Under these conditions, there may be some benefit to suppressing the activity of geniculate cells carrying weak responses or only spontaneous activity (for the condition when an eye is closed). The authors may have other examples, but some discussion about whether the conditions needed for non-dominant eye suppression occur naturally would be useful for the reader.

7. The introduction and discussion elude to the idea that contrast-dependent binocular interactions in the LGN could have implications for known psychophysical phenomena, such as interocular suppression. It would be helpful if the authors were more specific about what forms of suppression they are think about. Is binocular rivalry one of the phenomena they are considering? Whatever they might be, citations are needed.

Additional items

1. Details about the size of the fixation window should be provided.

2. Line 54, “or receive inputs from binocular neurons in other brain structures.” please add citation here.

3. Lines 60 and 61, please resolve the apparent conflict between the two paragraphs: the first emphasizing the differences between cats and monkeys and the second their similarities.

4. Line 141, “Infrared-light sensitive cameras... were used", it is not clear if one or two cameras were actually employed and whether the two eyes were monitored simultaneously or not.

5. Line 145, add some detail on the visual task for binocular fusion.

6. Line 222, why discarding data for rates exactly less than 1.9 spikes/s?

7. Line 250, please indicate the MATLAB smoothing function used.

8. Line 301. Please be specific about the number of units included from Monkey I34 (is it 15?).

9. Just a technical question. For many recordings, the monitor refresh was extremely low (60 and 85 Hz) which magnocellular neurons can follow. Why was such a low refresh used?

10. Line 392, “One prior study of LGN units in anesthetized” macaques, instead of “primates” (to make clear that this study refers to macaques and not marmosets).

11. Line 397, “We did not test this condition in the present study", refers to the monocular stimulation of the non-dominant eye in Rodieck and Dreher’s (1979) study? Clarify.

12. Figure 1A, add size calibration bars (in degrees of visual angle).

13. Figure 1C, please enumerate a few electrode contacts to make it easier to associate them to their respective spike density functions.

14. Figure 2A, it was not clear to me what is being shown in the sequence of three raster plots for trial 10. Does it merely refer to how an autocorrelation is built? If this is the case, it would be preferable to remove it from the figure for simplicity. Moreover, I do not see the necessity of the diagrammatic linking arrows to the figure’s right.

15. Figure 2C, the average correlogram exhibits a high-frequency modulation around the central bin. Are these fast oscillations in the spiking responses entrained by the monitor refresh (85 Hz)?

16. Figure 3A, PSD shows a dual-frequency component for the M cell. Is this second higher frequency peak just a harmonic of the temporal frequency of the gratings? Are harmonic peaks more prevalent specifically for the M cells? Why?

17. Figure 3C legend, please indicate that the expected functions are derived from published data.

## Author Response

Dear Dr. Löwel and reviewers,

We are grateful for your helpful comments regarding our manuscript “Binocular suppression in the

macaque lateral geniculate nucleus reveals early competitive interactions between the eyes.” Please find

below a point-by-point reply to those comments. We believe that these revisions improve the quality of

the manuscript, and we hope that you find it now fit for publication in eNeuro.

Major points

1. This study assumes that the experimental viewing conditions allowed for singular vision (the

stimuli presented dichoptically were fused). But do we have evidence that this actually

happened? I have the impression that the authors avoided the problem of rivalry, even though

recognizing that the suppression was more significant when the contrast discrepancies between

the two eyes were high. In this context, it would be most interesting if the authors could show

data for grating stimuli differing both in contrast and orientation. In a condition of rivalry, are the

contrast effects the same? This question is particularly relevant considering that the binocular

interactions in the LGN are possibly mediated by layer VI binocular cell inputs from the cortex.

The reviewer is correct that, despite great interest in binocular rivalry, we were exclusively focused on

conditions that are known to induce binocular fusion in this study. Specifically, we presented gratings of

identical spatial position, orientation, spatial frequency and phase that only differed in interocular

contrast. Interocular contrast differences alone are insufficient to induce binocular rivalry [1-5].

We agree that it is an interesting question whether there are correlates of binocular rivalry in the LGN.

Prior work in macaques suggest that LGN neurons do not alter their responses to rivalrous stimuli [6], yet

several human fMRI studies suggest otherwise [7, 8]. However, it takes several years to train macaques to

accurately report their perception during binocular rivalry [9]. We thus believe that an investigation of

binocular rivalry in the LGN lies beyond the scope of the current study. We added a section to the

Discussion section explaining this rationale (see lines 457-469).

References

1. Ding, J., and Sperling, G. (2006). A gain-control theory of binocular combination. Proc Natl Acad Sci U S

A 103, 1141-1146.

2. Huang, C.B., Zhou, J., Zhou, Y., and Lu, Z.L. (2010). Contrast and phase combination in binocular vision.

PLoS One 5, e15075.

3. O’Shea, R.P., and Crassini, B. (1982). The dependence of cyclofusion on orientation. Percept Psychophys

32, 195-196.

4. Kertesz, A.E., and Jones, R.W. (1970). Human cyclofusional response. Vision Res 10, 891-896.

5. Ding, J., and Levi, D.M. (2017). Binocular combination of luminance profiles. J Vis 17, 4.

6. Lehky, S.R., and Maunsell, J.H. (1996). No binocular rivalry in the LGN of alert macaque monkeys.

Vision Res 36, 1225-1234.

7. Wunderlich, K., Schneider, K.A., and Kastner, S. (2005). Neural correlates of binocular rivalry in the

human lateral geniculate nucleus. Nat Neurosci 8, 1595-1602.

2

8. Haynes, J.D., Deichmann, R., and Rees, G. (2005). Eye-specific effects of binocular rivalry in the human

lateral geniculate nucleus. Nature 438, 496-499.

9. Leopold, D.A., Maier, A., and Logothetis, N.K. (2003). Measuring subjective visual perception in the

nonhuman primate. Journal of Consciousness Studies 10, 115-130.

2. It seems that the authors gave a lot of importance to fixational eye movements as a

confounding factor in their experiments (lines 305, 456). It was difficult to follow this argument in

Figure 2A. It would be useful to see the eye traces juxtaposed to the responses in Figure 2A to

examine the magnitude of these effects. How can 1 to 2 micro-saccadic events (1-second

stimulus duration) falsify response latency estimation (line 456)?

We agree with the reviewers that one to two microsaccades within a trial will unlikely impact response

latency estimation. Re-visiting the eye movement data following the reviewers’ suggestion, we observed

that there was substantial variability in the initial fixation position from trial-to-trial, which shifted the

position of the grating stimulus over the RF. Below is a plot of the initial horizontal (x) and vertical (y)

gaze positions across trials from the session shown in Figure 2A, calculated as the mean between 0 and 15

ms post-stimulus onset. We modified the language on lines 323-333 in the Results and 511-514 in the

Discussion, and include a description of the range of the initial fixation position for the session shown in

Figure 2A.

3. We tend to think that the total size of the analyzed data (N= 56 units) is relatively small. This

is true, especially if we consider that one of the main objectives of this study was a differential

analysis across cell types (M, P, K). From a single monkey, only 15 cells were taken (across 13

recording sessions). In total, only six cells of type K were analyzed. Are these samples not too

small given that linear multi-electrode matrices with 24 or 32 contacts were used for the

recordings?

The reviewers are correct that we used linear multicontact arrays with 24 or 32 contacts for the

recordings. The primary benefit of using these probes for these experiments was that we could monitor

the multiunit hash online across contacts on the probe. This online monitoring is useful to distinguish the

LGN from nearby visual structures, such as the pulvinar.

However, because of the shape of the LGN, the receptive field locations across the probe rarely aligned.

We opted to collect data using a stimulus that covered the RF of the best isolated spikes on the probe. If a

3

nearby channel happened to have an RF center that overlapped or nearly overlapped (< ∼3 degrees

separation) with the best channel, we adjusted the stimulus so that it covered both RF centers, and

collected data for those channels simultaneously. Otherwise, we were unable to take advantage of most of

the contacts on the probe for single unit data. We now include a note about this limitation in the Results

section (see lines 315-321).

4. In the Discussion, line 445: Is interocular suppression used as a gain control mechanism

across eye inputs? If these effects are seen in the LGN, why the authors think that they arise in

the visual cortex? Please clarify this point.

We thank the reviewer(s) for bringing this ambiguity to our attention. We now elaborate on interocular

gain control in the LGN and visual cortex in the Discussion (see lines 477-487).

5. Line 434. In this paragraph, the authors discuss their results in terms of competition between

the two eyes, which is a reasonable topic for discussion. Another interpretation of the results

that the authors should consider is whether they have identified a means to reveal details about

the circuits that mediate gain control. LGN neurons are known to have contrast gain control that

is augmented relative to their retinal inputs, indicating the involvement of extraretinal

mechanisms. Results from the current study could be interpreted as evidence in support of

extraretinal mechanisms for gain control, as well as for the idea that extraretinal gain control

circuits are binocular. These ideas could be included to broaden the implications of the study’s

results in the Discussion.

We thank the reviewer(s) for providing this important insight. We agree that is possible that the effects we

report in this manuscript come about through binocular extraretinal inputs mediating gain control, and we

now include text on this possibility in the Discussion (see lines 487-492).

6. While it is difficult to think of situations where a stimulus in a specific region of space would

have different contrasts for the two eyes, it could occur if the eyes were not aligned or if one eye

is closed. Under these conditions, there may be some benefit to suppressing the activity of

geniculate cells carrying weak responses or only spontaneous activity (for the condition when

an eye is closed). The authors may have other examples, but some discussion about whether

the conditions needed for non-dominant eye suppression occur naturally would be useful for the

reader.

We now include a description of when interocular contrast differences occur in natural vision, as well as a

discussion on how the results shed light on the nature of interactions between the two eyes, even if the

extreme examples shown here are unlikely in natural vision (see lines 493-502).

7. The introduction and discussion elude to the idea that contrast-dependent binocular

interactions in the LGN could have implications for known psychophysical phenomena, such as

interocular suppression. It would be helpful if the authors were more specific about what forms

of suppression they are think about. Is binocular rivalry one of the phenomena they are

considering? Whatever they might be, citations are needed.

Binocular rivalry is one case of interocular suppression that we now discuss on lines 456-468. We also

now discuss other instances of contrast-dependent binocular interactions on lines 477-502.

4

Additional items

1. Details about the size of the fixation window should be provided.

Please see this detail add on line 216.

2. Line 54, “or receive inputs from binocular neurons in other brain structures.” please add

citation here.

Please see citation add on lines 54-55.

3. Lines 60 and 61, please resolve the apparent conflict between the two paragraphs: the first

emphasizing the differences between cats and monkeys and the second their similarities.

Please see modifications to these paragraphs, now in one single paragraph on lines 57-68.

4. Line 141, “Infrared-light sensitive cameras... were used", it is not clear if one or two cameras

were actually employed and whether the two eyes were monitored simultaneously or not.

Please see the clarification on two cameras used in the majority of sessions on lines 57-68.

5. Line 145, add some detail on the visual task for binocular fusion.

Please find a description of the task for binocular fusion on lines 149-158.

6. Line 222, why discarding data for rates exactly less than 1.9 spikes/s?

In preprocessing the data, one of the first steps we took was to exclude clusters from KiloSort that were

unstable or had very low firing rates. Following this step, we checked clusters further for stability, visual

responsiveness, typical contrast responsive curves as detailed in the Methods. While the value may seem

arbitrary, there were four clusters in the sample with a mean firing rate across the entire file between 1.9-

2.0 spikes/s. We decided to lower the criterion from 2.0 to 1.9 spk/s in order to avoid reducing the sample

further. These clusters had to meet other criteria regarding stability and visual responsiveness listed in the

Methods to be included in further analysis.

7. Line 250, please indicate the MATLAB smoothing function used.

Please find that the shape of the kernel (normal) used for the non-parametric smoothing is now included

on lines 262-265.

8. Line 301. Please be specific about the number of units included from Monkey I34 (is it 15?).

Please see the number of units and sessions for Monkey I34 on line 315.

5

9. Just a technical question. For many recordings, the monitor refresh was extremely low (60

and 85 Hz) which magnocellular neurons can follow. Why was such a low refresh used?

We appreciate this question as it prompted us to look at the refresh rate of the monitor for each unit. In

actuality, the refresh rate was set to 60 Hz for only one unit. Most units (n = 50 units) were collected with

a refresh rate of 85 Hz on a CRT monitor, and the rest (n = 15 units) were collected using VPixx monitor

with a reported refresh rate of 120 Hz. At the start of the recordings, we were limited using slow refresh

CRTs that had been used by neighboring labs, as this was still more desirable than using a standard LCD

monitor with slow rise and fall times. When grant funding allowed, we purchased a VPixx monitor with a

higher refresh rate. We now include these values in the Methods section on lines 131-133.

10. Line 392, “One prior study of LGN units in anesthetized” macaques, instead of “primates” (to

make clear that this study refers to macaques and not marmosets).

This language has been adjusted on line 414.

11. Line 397, “We did not test this condition in the present study", refers to the monocular

stimulation of the non-dominant eye in Rodieck and Dreher’s (1979) study? Clarify.

Yes, the reviewer is correct that “condition” refers to stimulation of the non-dominant eye alone. We now

clarify this point on line 418-419.

12. Figure 1A, add size calibration bars (in degrees of visual angle).

We now indicate in the legend that the schematic in Figure 1A is not drawn to scale, and we indicate the

size of the fixation cross in the Methods section on line 217.

13. Figure 1C, please enumerate a few electrode contacts to make it easier to associate them to

their respective spike density functions.

We now moved the labels for the electrode contacts to the right-hand side of the responses and include

more labels.

14. Figure 2A, it was not clear to me what is being shown in the sequence of three raster plots

for trial 10. Does it merely refer to how an autocorrelation is built? If this is the case, it would be

preferable to remove it from the figure for simplicity. Moreover, I do not see the necessity of the

diagrammatic linking arrows to the figure’s right.

The reviewer(s) is correct that the sequence of plots is demonstrating how the auto-correlation is built. To

keep things simple, we removed this diagram from the figure.

15. Figure 2C, the average correlogram exhibits a high-frequency modulation around the central

bin. Are these fast oscillations in the spiking responses entrained by the monitor refresh (85

Hz)?

6

We double-checked the response of the example unit shown in Figure 2C. Below is a plot of the

oscillatory power (based on the IRASA method; Wen & Liu et al., 2016) of the autocorrelation of the

spikes, with frequency extend out to 130 Hz on the x-axis. There is a peak in the oscillatory power at

∼110 Hz. The reported refresh rate of the monitor on the day of this recording session was set to 120 Hz.

We suspect that the peak in power near 110 Hz is due to the monitor refresh, perhaps because the refresh

rate was internally adjusted to 110 Hz by the monitor. To validate the results on the left, we show a

histogram of the inter-spike intervals for this unit. There is a peak in the distribution where one would

expect 110 Hz modulation (∼9.09 ms).

16. Figure 3A, PSD shows a dual-frequency component for the M cell. Is this second higher

frequency peak just a harmonic of the temporal frequency of the gratings? Are harmonic peaks

more prevalent specifically for the M cells? Why?

We thank the reviewer for calling on us to explain this data point. The prominent 8 Hz modulation occurs

specifically for M units for the LM-opponent stimulus for a specific reason. M neurons receive input from

retinal ganglion cells that sum inputs from L and M cones (see Lee, Martin, Valberg, 1989b).

Consequently, the 1 cyc/{degree sign} stimulus drifting at 4 Hz stimulates the RF center of M neurons 8 times per

second because two phases of the sinusoidal grating-that corresponding to L and that to M-stimulate

the RF. We now include a brief explanation of this in the Figure 3B legend.

17. Figure 3C legend, please indicate that the expected functions are derived from published

data.

We now include this point in the Figure 3C legend and point the reader to the Results section where we

include citations related to these expected results.
